# Validation and evaluation of school-based mental health literacy training program "*The Guide Cymru*" in Iranian adolescent students aged 13–15: study protocol

**DOI:** 10.1186/s13690-024-01257-w

**Published:** 2024-03-08

**Authors:** Batool Zeidabadi, Mahsa Khodayarian, Reza Sadeghi, Sara Jambarsang, Mina mohseni

**Affiliations:** 1 Department of Health Education & Health Promotion, International Campus, Shahid Sadoughi University of Medical Sciences, Yazd, Iran; 2 Department of Health Education & Health Promotion School of Public Health, Social Determinants of Health Research Center, Shahid Sadoughi University of Medical Sciences, Yazd, Iran; 3 Department of Public Health, Sirjan School of Medical Sciences, Sirjan, Iran; 4 Center for Healthcare Data Modeling, Departments of Biostatistics and Epidemiology, School of Public Health, Shahid Sadoughi University of Medical Sciences, Yazd, Iran; 5 Sirjan School of Medical Sciences, Sirjan, Iran

**Keywords:** Mental health literacy, Randomized trial, Adolescent, School, Knowledge, Help seeking, Stigma, Mental health

## Abstract

**Background:**

Schools are an ideal setting for enhancing mental health literacy, a crucial strategy for improving adolescents’ mental health knowledge and attitudes and promoting help-seeking. "*The Guide Cymru"* program is an adaptation of the mental health literacy program*" The Guide"* that was developed in Canada. The program will be culturally adapted for 13- to 15-year-old Iranian adolescent students in the first secondary schools.

**Methods:**

In this randomized controlled trial, using the stratified random sampling procedure, the whole eighth and ninth grade student body (aged 13 to 15) from Sirjan City's first secondary schools will be included in the study. Twenty first secondary schools will be randomly assigned to one of two groups: control or intervention. Finally, 562 students and 40 teachers will participate in the research. The tools of mental health literacy scale (MHLs), mental health general knowledge and attitudes related to mental disorders/illnesses are employed in this study.

**Discussion:**

This trial aims to be to explore whether "*The Guide Cymru"*, a mental health literacy program offered to students as part of the school curriculum, can decrease the stigma associated with mental health and promote help-seeking behaviors among students.

## Background

Adolescent mental health has received a great deal of attention and has been brought up as a public health concern [1]. Mental health issues are estimated to affect 10 to 20 percent of adolescents and children [2]. Adolescents' performance, development, and wellbeing alike can all be influenced by poor mental health [3]. if neglected, it may progress into adulthood and cause physical and mental issues [4]. Despite the high prevalence of mental health problems among adolescents, proper help-seeking for such concerns is uncommon [3]. In fact, there is a need to enhance help-seeking in order to reduce the rising prevalence of mental health problems among adolescents as well as to promote mental health literacy as a key strategy for help-seeking promotion among adolescents [5, 6]. Mental health literacy is an evolving concept that has been conceptualized in various ways from the time Jorm Jorm, Korten [7] coined the term in 1997 [8, 9]. In past years, mental health literacy has shifted from focusing on mental illness and risk factors to becoming an asset for health [10]. A more recent definition of MHL includes four components: [1] understanding how to obtain and maintain good mental health; [2] understanding mental disorders and their treatments; [3] decreasing the stigma associated with mental disorders; and [4] enhancing help-seeking efficacy (i.e., knowing when and where to obtain evidence-based mental health care and possessing the competencies to enhance self-care) [11].

Adolescence is a time when significant increases in the diagnosis of mental disorders are observed. In the survey of psychiatric disorders among children and adolescents in Iran (2016), the general rate of occurrence of psychiatric disorders was reported to be 10.55% [12]. In 2019 national survey of children and adolescents, which looked at the prevalence of mental diseases; 22.31% of Iranian children and adolescents had at least one psychiatric disorder [13]. Therefore, an increasing trend was observed in the prevalence of mental disorders among Iranian children and adolescents. Psychiatric disorders grouped in the category of anxiety disorders with a prevalence of 14.13% were the most frequent mental disorders among all children and adolescents; this category included the separation anxiety (5.34%), special phobia (4.84%), obsessive compulsive disorder (3.48%), agoraphobia (2.86%), general anxiety (2.57%), social phobia (1.8%), posttraumatic stress disorder (0.5%), and panic disorder (0.2%) [13].

Really, the high prevalence of mental disorders justifies the special attention of health systems to the mental health status of children and adolescents. Therefore, providing the adolescents with skills to manage their mental health as well as assisting to others, including peers is critical [14]. Inasmuch as adolescents frequently prefer to seek assistance from their peers for mental health issues, mental health education should be an ongoing component of the school curriculum [2]. Considering the vital role of education in mental health literacy, schools will provide an appropriate setting for educating vulnerable people about their mental health literacy. The reason is that adolescents spend the majority of their time in school, and education is inextricably associated with health [15]. Furthermore, a teacher with a strong and persuasive personality can play an important role in fostering students' mental health literacy by helping them to have the courage and confidence to discuss their mental health with a trusted one [3]. Evidences indicate that schools are ideal venues to address and enhance various aspects of mental health literacy in both students and teachers [16]. The results of studies in Canada [14], Cambodia and Vietnam [17], Wales [3] and Nicaragua [18] regarding the effectiveness of *The Guide* program in improving mental health literacy in high school students showed that the mental health literacy and the knowledge and attitude of students were increased after the implementation of the program in the classroom. In addition, the knowledge and attitude of mental health literacy among teachers increased significantly [18].

Nonetheless, health professionals and policymakers have recognized the significant role of schools in addressing the mental health requirements of adolescents [19]. Therefore, a mental health literacy program provided as part of the school curriculum to students can reduce the stigma associated with mental health and increase help-seeking behaviors for mental health concerns [20].

### The Guide Cymru

The school-based mental health literacy program, adapted and modified from the original Canadian handbook [21], was designed to improve the knowledge and attitudes of 13–15-years-old high school students about mental health. *The Mental Health and High School Curriculum Guide (The Guide)* is a school-based mental health literacy resource developed by mental health experts, educators and the Psychiatric Unit of Health Canada [22].

The school-based mental health literacy program guide is the only scientific mental health curriculum resource demonstrating that students' mental health literacy can be enhanced through teacher training programs as well as their applications in various programs. It is noteworthy that this edition of *The Guide* has supplanted all previous editions and has been rewritten with new contents [6]. *The Guide* has been settled to help students enhance their knowledge of mental health and is used in the first year of secondary school (aged 13 to 15). Usually, mental disorders onset during adolescence; so it is crucial that adolescents have the required knowledge, attitude, and skills to assist themselves and others when necessary.

Mental health literacy includes four components of 1) understanding how to obtain and maintain good mental health; [2] learning about mental disorders and their treatments; [3] decreasing the stigma associated with mental disorders; and [4] enhancing help-seeking efficacy (i.e., knowing when and where to obtain evidence-based mental health care and possessing the competencies to enhance self-care).*The Guide* assists students in achieving success in each of these areas. It should be noted that *The Guide* as mental health literacy resource is taught in 8 to 12 class hours using a combination of instructional methods, group discussion, group activities, self-directed learning, and videos.

The *Guide Cymru* covers six areas including: 1) Understanding mental health and mental illness, 2) Stigma of mental illness, 3) Information about specific mental illnesses, 4) experiences of mental illness, 5) seeking and receiving support, and 6) the importance of positive mental health [13]. Furthermore, *The Guide* includes material for teacher self-study, which provides them with an in-depth comprehension of the subject matter they teach. The important point is that teachers who utilize *The Guide* in their classes go through a training session to get acquainted with the material.

## Objectives

### Primary objective

The primary purpose of this study is translation and cultural adaptation of the school-based mental health literacy program guide as well as validation of the *Guide Cymru* program in Iranian adolescent students aged 13–15. Also, the study deals with with the implementation and evaluation of program.

### Practical objective

Following the translation and validation, *The Guide* will be utilized as a national protocol for the mental health curriculum in the first secondary level. Additionally, *The Guide* will be utilized by teachers who have been educated in its usage to raise the mental health literacy of first secondary students aged 13–15.

## Methods/design

### Design of the study

The present study is a randomized controlled trial with the aim of validating and evaluating the *Guide Cymru* school-based mental health literacy training program in Iranian adolescent students aged 13–15 in 2023.

### Population and sampling

The study population includes all adolescents in grade 8th and 9th year of secondary schools (13 to 15 years old) using stratified random sampling from Sirjan city; a city in Kerman province in the southeast of Iran. Using the effect size and primary data of Nguyen, Dang [17] study and considering the first type error at the level of 0.05 and the power of 0.80, the sample size was calculated to be 281 students in each of the intervention and control groups. Finally, 562 students of the first secondary schools of Sirjan city will be included in the study.

$${n}_{1}={n}_{2}=\frac{\left({S}_{1}^{2}+{S}_{2}^{2}\right){\left({Z}_{1-\frac{a}{2}}+{Z}_{1-\beta }\right)}^{2}}{{\left(\overline{{X }_{1}-\overline{{X }_{2}}}\right)}^{2}}$$

After receiving the list of first secondary schools from the Department of Education, in order to prevent the effect of the intervention and based on the three geographic regions of Sirjan, a total of 20 first secondary schools will be randomly divided into two groups of control and intervention. This number of schools was determined based on the assumption that each school has a minimum of 28 students to be included in the study. According to the procedure for implementing the intervention, two teachers (a health coach and an educational coach) will be chosen from each school. Twenty teachers from each of the two groups (intervention and control) will take part in the study, for a total of 40 teachers. If a teacher expresses unwillingness, will be replaced by another teacher.

## Recruitment and selection

### Inclusion and exclusion criteria

#### Eligible schools

The public schools of Sirjan city, wishing to implement the educational intervention of the mental health literacy program for the grades of 8th and 9th, and introduce two teachers to the two-day educational program as well as provide the essential conditions for their involvement will be included. Eligible students are also students in the age group of 13 to 15 years, studying in the 8th and 9th grades in a public school in Sirjan.

#### Exclusion criteria for schools

Exceptional schools with students of learning disabilities or behavioral problems as well as non-profit schools.

After receiving the code of ethics, permission and letter of introduction from the Research Vice-Chancellor of Shahid Sadougi University of Medical Sciences, Yazd, the necessary coordination was done with the education department of Sirjan city. In the following, the names of the instructors engaging in the study were determined and after describing the objectives of the study and expressing willingness, they were invited to collaborate as facilitators in this study. The research team then invited all included teachers to attend a two-day training course for the mental health literacy program. The training course was held with the presence and supervision of a psychiatric specialist to ensure the quality of the course and the specialization of mental health issues.

#### Design of the study

**Fig. 1:**
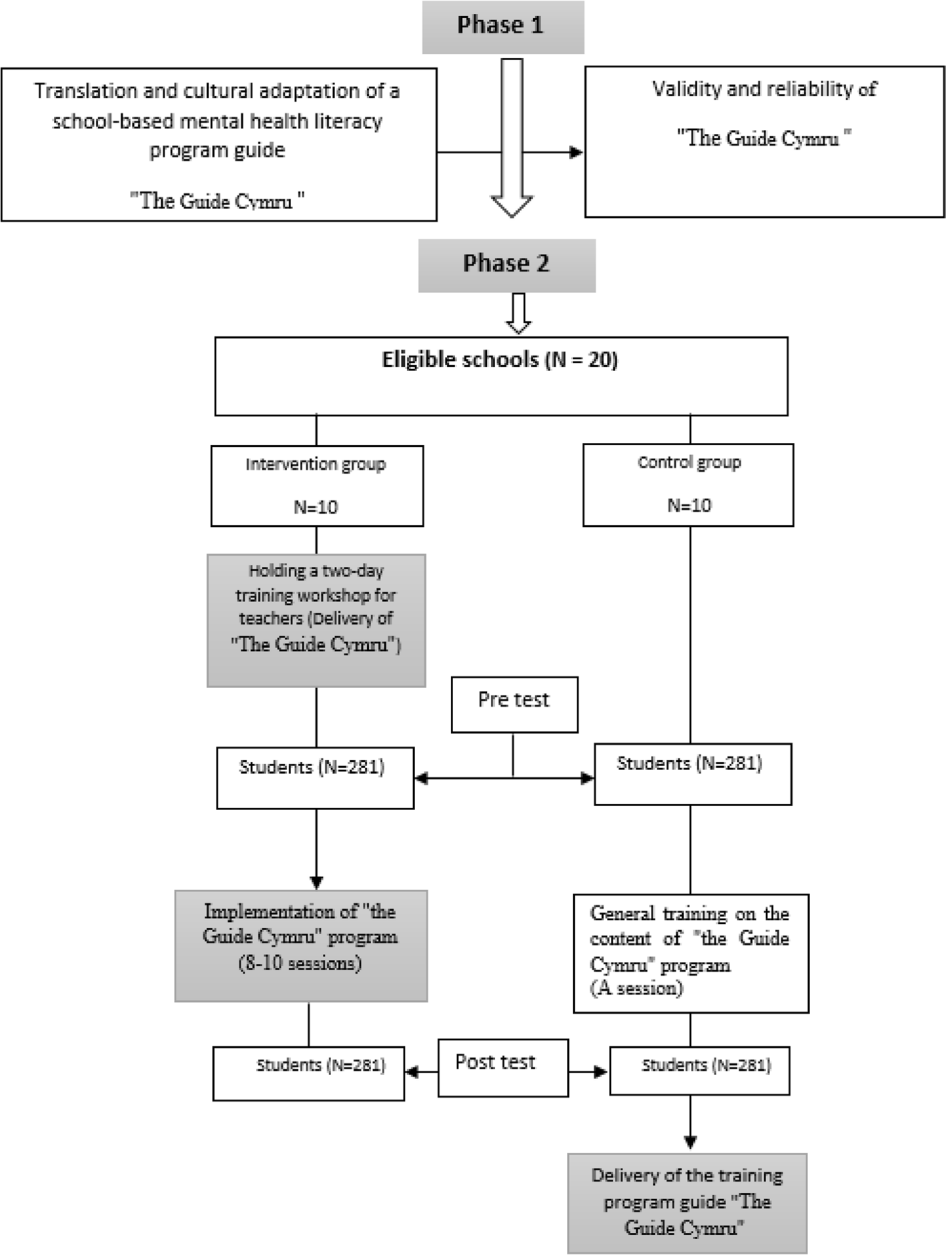
Procedure of school-based mental health literacy program *The Guide Cymru*

In this study, the validation and evaluation of school based mental health literacy educational program, the Guide Cymru will be conducted on adolescent students aged 13 to 15 according to Fig. 1. Shows the procedure to be followed.

#### Study phases

Followings are the procedures for conducting the study in two phases:

Phase 1: Translation and cultural adaptation of the mental health literacy program* "The Guide Cymru".*

- A) Translation and cultural adaptation of *"The Guide Cymru".*
  - b) Accreditation of the school-based mental health literacy training program "The Guide Cymru".
  - c) Suitability and readability assessment of materials (SAM & RAM).
  - d) Validity and reliability of general mental health knowledge and attitudes related to mental disorders/illnesses.
    - Phase 2: Implementation of the educational program and evaluation of the school-based mental health literacy program *"The Guide Cymru".*
      - Step 1) Pre-test.
    - Phase 2) Empowering teachers in mental health literacy based on school-based mental health literacy program *"The Guide Cymru".*
      - Step 3) Implementation of an educational program for students based on school-based mental health literacy program *"The Guide Cymru".*
      - step 4) Evaluation of the impact of school-based mental health literacy program *"The Guide Cymru".*

## Implementation of phase 1

### Translation and cultural adaptation of Mental Health Literacy Guide *"The Guide Cymru"*

Prior to start the study, we will communicate with *"The Guide Cymru"* developers to get their permission to translate it into Persian. Then, in the first step, the mental health literacy guide will be translated into Persian as well as culturally adapted according to the guide of Beaton, Bombardier [23] guide. In the next step, the form and content Validity and reliability of general mental health knowledge and attitudes related to mental disorders/illnesses which are available in *The Guide* are tested.

### A) Translation and cultural adaptation of *"The Guide Cymru"*

After receiving written permission from the guide's primary developers via email, the translation process will commence. The translation approach used in this study is based on the IQOLA standard procedure, which comprises translation phases, translation quality evaluation, reverse translation, and a comparison of the English and Persian versions by the guide's primary developers [24]. The first step is to translate the guide. Initially, two Persian native speakers who are both fluent in English will translate the guide into Persian without knowing one another. Then, both translations will be combined and the finest terms will be selected to create a single version. The second phase is the reverse translation, in which the Persian translation will be converted into English by two native speakers of the original language (English) who are unaware of each other as well as unaware of the original version of the scale. Then, the two versions are combined, compared, and the finest terms are chosen to form a single version. The third step is the collective review, which examines and revises the final translation in a concentrated group-discussion session with the consultation of subject-matter experts(People who have sufficient expertise in the field of tool making and its localization, as well as familiarity with the category of mental health, including clinical psychology, psychiatry, educational sciences, and health education and health promotion) for improved comprehension and in accordance with the original guide.

Finally, the final English version will be emailed to the principal developer for final approval. Regarding the conformity of the translated version with the original version, their opinions were sought and the conceptual consistency and conformity between the original version and the Persian-to -English translated version were confirmed.

### B) Validity of the school-based mental health literacy program *"The Guide Cymru"*

Educational tools must be highly applicable in real-world applications. Van Der Vleuten [25] developed five characteristics of measurement tools, namely validity, reliability, feasibility, acceptability, and educational impact. Each of these features is important to users. Tools must be valid and reliable. An unreliable instrument will not be able to track the success of training over time in a consistent and error-free manner. Acceptability considers the tool's suitability from the users' point of view. This feature not only entails measuring construct validity but also results in the feasibility of instruments and educational impact [26]. Users (students, instructors) need to know that the training program has a sufficient amount of content and that internal consistency (reliability) increases as the number of training items increases [27]. Van Der Vleuten [25]underlines the necessity of assessing all of these factors when selecting the proper instrument for the proper purpose. For example, if the outcomes of the curriculum guide are to be utilized at higher levels, it must have high reliability, educational effect, and acceptability in order to increase students' mental health literacy. The school-based mental health literacy training program validity will be determined using face validity, content validity, feasibility, acceptability, and educational impact.

### Feasibility, Acceptability, and Educational Impact

Feasibility, acceptability and educational impact of *The Guide Cymru* will be examined through a checklist of four questions with a bipolar range of excellent/completely applicable to very weak/not applicable at all, including (overall evaluation of the program, improvement of teachers' mental health literacy, improvement of students' mental health literacy, usability of educational program) in the presence of twelve mental health experts.

### Face validity

In face validity, the appearance of the tool is examined by the target group. If a tool has face validity, more motivation will be created in the target group to complete it. To assess the qualitative face validity, face-to-face interviews will be performed with ten people from the target group (five teachers and five students). The main researcher, who has complete control over the educational program, will conduct this interview. People are asked to read the educational program's guide and express their understanding of the program's educational content to the researcher. They will also be asked about the level of difficulty, i.e. difficulty in comprehending phrases and words, the degree of relevancy, i.e. the potential incompatibility of the educational program's contents from the respondents' perspective, The degree of ambiguity, i.e. the likelihood of phrase misinterpretation or semantic failure. Also, this group's recommendations will be followed, and this process will continue until people's understanding of the Training program guide is simplified and no new adjustments are requested [28].

### Assessing Content Validity Using A Qualitative Approach

In order to qualitatively assess the content validity in this study, twelve professionals with experience and education in mental health, health education, health promotion, and educational sciences will be requested to assess the guide for grammar, suitable terminology, and module content layout.

### Content Validity Ratio (CVR)

The content validity ratio is determined by gathering ratings from a group of experts, including a psychology expert, a health education and promotion expert, and an educational science expert. They are asked to assess the necessity of each item on a three-part Likert scale (necessary, beneficial but not necessary, unnecessary). In accordance with the Lawshe method and the formula below, every model demonstrates a satisfactory CVR. CVR = (Ne-N/2)/ (n/2).

### Content Validity Index (CVI)

The content validity index (CVI) will be checked using the Waltz and Basel methods. Experts will use a 4-part Likert scale to judge the "relevance" and "clarity" of each question. Then, the value of CVI will be determined by dividing by dividing the number of experts who agree with the question (on a Likert scale, scores of 3 and 4 are regarded agreeable) by the total number of experts [29]. Afterwards, the average CVI score of relevance and clarity associated with each question will be calculated and then reported. Based on the CVI score, Hyrkas et al. proposed a score of 0.79 or higher to approve each question [30].

### C) Suitability Assessment of Materials of (SAM) and Readability Assessment of Materials (RAM)

In order to check the suitability as well as readability of educational content of mental health literacy program guide, SAM and RAM will be used with the first one checks the suitability and the other one checks the readability of materials [31].

### Suitability Assessment of Materials of (SAM)

SAM evaluates written materials according to 22 criteria organized into six categories: "Content," "Required Literacy Level," "Graphics," "Layout and Typography," "Learning Stimulation and Motivation," and "Appropriateness." Each factor is scored as excellent (2 points), suitable (1 point), or non-suitable (0 points). Not applicable are factors that do not apply to a given material. The maximum possible score is 44, but 2 points are subtracted for factors that are not relevant. The overall SAM score is made by adding up the scores for each item. Then, this score is turned into a percentage by dividing the total SAM score by the total possible score for that particular subject. This material is classified as non-suitable (0%-39%), suitable (40%-69%), or excellent (70%-100%) [31].

### Readability Assessment of Materials (RAM)

RAM evaluates the difficulty of reading an educational media into three categories: specialized content (score range 0–6), misspellings (score range 0–6), and typographical mistakes (score range 0–6). The range of scores in media readability evaluation is 0 to 18, with a score of more than 10 being satisfactory [31].

### The guide cymru's teacher evaluation

The school-based mental health literacy program will be evaluated by 20 trained teachers using the evaluation checklist that will be designed. In this way, the teacher will complete the evaluation checklist following each training session. On the basis of the teachers' feedback, the curriculum will be evaluated.

### Reliability and Validity of General Mental Health and Attitudes to Mental Disorders/Illnesses

At this point, the tool will be translated in compliance with IQOLA standards after obtaining the developer's permission [24]. Two English language professionals translate the instrument into Persian, and then the study team's researchers and mental health experts review, modify, and make the necessary changes. The translation will then be back translated into English by two experts. Following assessment by the study team's researchers, the English translation created at this step will be sent to the tool maker via email to validate the correctness of the translation and the right understanding of the tool's items and after the approval, the Persian version of the instrument will be psychometrically assessed at this stage [30].

### Determining face validity:

The questionnaire's face validity will be assessed using two qualitative and quantitative methodologies. To examine the face validity qualitatively, 10 experts in the fields of health education and promotion, psychology, psychiatry, and biostatistics will assess the degree of difficulty, suitability, and ambiguity and make any required modifications. Furthermore, the item impact method will be utilized to test the face validity quantitatively. As a result, the 5-valued Likert scale runs from 5 to 1 point for each instrument item, with the possibilities of very important, rather important, moderately important, somewhat important, and not important at all. In addition, ten experts will be asked to assess the significance of each item for the provided structure based on their past knowledge. Using the following formula, the impact score for each item is then calculated. All items greater than 1.5 will be regarded as acceptable.

$$\mathrm{Impact}\;\mathrm{Score}\:=\:\mathrm{Frequency}\;(\%)\:\times\:\mathrm{Importance}$$

I

The proportion of respondents who gave the item a score of 4 or 5 represents frequency, and the average importance score based on a 5-point Likert scale represents importance. If the impact score exceeds 1.5, the item is considered eligible for further study and is kept [29].

### Content Validity

The content validity will be determined using two qualitative and quantitative methodologies. As part of the qualitative review of the content, the final translated version of the questionnaire will be presented to ten psychometric experts to provide the required feedback after a qualitative examination of the tool based on grammar, usage of relevant words, placement of phrases in the proper location, and appropriate scoring. Also, the Content Validity Ratio (CVR) and the Content Validity Index (CVI) will be used to assess the content validity quantitatively.

With CVR, the necessity of an item is assessed by professionals so that only the most significant and correct items are chosen and CVI evaluates the clarity, adequacy, or relevance of the items with respect to the researcher's objective are evaluated from the perspective of experts, which aids in designing the items in the most effective manner to measure the content. In order to determine the CVR, the group of experts is requested to assess as well as score each item's necessity based on the three-part Likert spectrum (necessary, useful but not necessary, and not necessary). It is therefore possible to ensure the selection of the most crucial and accurate content based on the scores obtained for each item.

In addition, to check the CVI, following distribution of the tool to ten psychometrics specialists, the score obtained from their opinions on the relevance, clarity and simplicity of each item will be calculated based on a four-part Likert scale. Therefore, it is possible to ensure the selection of the most relevant, simple and clear items. The minimum acceptable value for the CVI index is 0.79, and any item with a CVI index less than 0.79 should be eliminated [30].

### Tool reliability

The consistency of an instrument over time is referred to as its reliability, and the reliability test focuses on three aspects of internal consistency, stability, and equivalence. Internal consistency or stability is measured with Cronbach's alpha, and stability over time is measured with the test–retest method [32]. Two methods of internal consistency and stability will be employed in this study.

### Internal consistency/Cronbach’s alpha

Internal consistency, which indicates the agreement between the instrument's various dimensions, is a common method for determining its reliability. By splitting an instrument in half and comparing the two halves together, or by computing the Cronbach's alpha coefficient, the internal consistency of the instrument can be determined. In this study, Cronbach's alpha coefficient will be calculated after construct validity. The minimum acceptable level of alpha will be 0.7 [33].

### Stability/test–retest

Stability refers to obtaining identical scores from a group of individuals at two distinct time points and represents the degree of change over time. Thus, the stability of a tool is evaluated over time. Utilizing the test–retest method and calculating the intraclass correlation index, stability is evaluated. In the present study, in order to determine the consistency between the modules of the final program guide based on the test–retest criterion, the guide will be completed in two stages by 30 participants with an interval of two weeks. Then, using the intra-cluster correlation index (ICC), the scores obtained in these two steps will be compared [34]. The ICC rate ranges from zero to one, and the closer it is to one, the higher the reliability. According to China, a tool must have a reliability coefficient of at least 0.6 to be considered acceptable [35, 36].

### Phase II: Implementation of the training program and evaluation of the school-based mental health literacy program *"The Guide Cymru"*

- Step 1) Pre-test.
  - Phase 2) Empowering teachers in mental health literacy based on *the Guide Cymru.*
- Step 3) Application of a training program for students based on *the Guide Cymru.*
  - Phase 4) Evaluation of the impact of *the Guide Cymru'*s school-based mental health literacy program.

### Implementation of Phase II

#### Step 1) Pre-test

This component is intended to help the teacher with self-study before using the guide in the classroom. Participating in the pre-test assists in identifying areas where the teacher's knowledge base needs to be expanded. Following the participation, questions with incorrect answers are recorded. Then, the teacher's mental health literacy will be strengthened using the guide's educational resource. The post-test is administered following the completion of the teacher's mental health literacy empowerment. The pre- and post-test questionnaires for teachers will be mental health literacy and mental health general knowledge questionnaires. Questionnaires will be completed again two months later.

Teachers (control and intervention groups) will complete the Mental Health Literacy Scale (MHLS) and general mental health knowledge prior to the implementation of the program. Also, Students (control and intervention groups) complete questionnaires assessing mental health literacy, general knowledge of mental health, and attitudes toward mental disorders, in addition to a demographic information form. Lastly, the participants will decide when the best time is to hold the educational intervention classes.

### Phase 2) Empowering teachers in mental health literacy based on* the Guide Cymru*

The purpose of this component is to provide the teacher fundamental knowledge on mental health and mental diseases so they may utilize the resource more effectively in the classroom. The source guide is comprised of six modules including: 1) Stigma of Mental Illness, 2) Understanding Mental Health and Mental Illness, 3) Information About Specific Mental Illnesses, 4) Experiences of Mental Illness and the Importance of Family Communication, 5) Seeking Help and Support, and 6) The importance of positive mental health. Teachers in the intervention group will attend a two-day training session for the six-module school-based mental health literacy program as well as will get the he resources and content of the Persian-language version of the school-based mental health literacy program. Furthermore, The educational content of the Persian version of the website http://teenmentalhealth.org, which is a rich repository of original and supplementary educational materials based on six educational modules will be provided to teachers through available messengers.

### Step 3: Application of an educational program for students based on the Guide Cymru

Prior to the start of study, written consent will be obtained students' parents. Teachers will provide informed consent forms to parents. To keep the confidentiality and anonymity, consent forms will be gathered, and the study team will develop an identification code for each student, which will be completed by teachers. It is worth mentioning that this code will be will be used in student evaluation. Participants are also assured that their personal information will be kept strictly confidential. The evaluation is based on three questionnaires measuring mental health literacy, general knowledge of mental health, and attitudes toward mental disorders, allowing the teacher to assess the significant dimensions of mental health literacy. Before teaching the guide in the classroom, the pre-test will be administered; following the completion of the sixth module, the post-test will be administered again; and two months later, the questionnaires will be administered once more. Students will attend sessions on the specified day and time depending on the allocated groupings. Following the pre-test, a qualified teacher will lead an educational content session of the school-based mental health literacy program based on the six modules under the supervision of the researcher and psychologist. The Guide training consists of six modules taught sequentially over the course of eight to ten sessions. The modules are constructed in such a manner that each one is conducted in 60-min lessons. Among all modules of the Guide, the third module is the longest and most informative module, requiring more teaching time than the others. As a result, three 50-min intervals will be allotted to this module. In the control group, the general content of the Guide Cymru educational program will be presented by a psychological expert with the coordination of teachers and students. Finally, after the evaluation of the school-based mental health literacy program, teachers and pupils in the control group will receive the Guide Cymru program guide.

### Phase 4) Evaluation of the impact of Guide Cymru's school-based mental health literacy program

According to study of improvements in students’ mental health literacy with use of a mental health curriculum in Canadian schools [13], mental health literacy, general knowledge of mental health and attitudes related to mental disorders of students and teachers before and after the implementation of the educational intervention and with a 2-month follow-up were evaluated and the results will be compared before, immediately after, and two months after the educational intervention. By comparing a student's pre-test, post-test, and two-month post-test scores, this method of evaluation enables a precise determination of student's learning.

### Data Collection Method

In this study, data will be gathered through questionnaires on mental health literacy, general knowledge of mental health, and attitudes toward mental disorders, as well as demographic information.

### Questionnaire 1) Mental Health Literacy Scale (MHLS)

The Mental Health Literacy Scale (MHLS) was introduced by O'Connor and Casey in 2015 and includes six attributes of the ability to recognize specific disorders, knowledge of professional help available, knowing how to seek mental health information, knowledge of self-treatments, knowledge of risk factors and causes, and attitudes that promote recognition and appropriate help-seeking [7]. These features were examined and compared to the findings of Jorm [37] and Griffiths, Christensen [38] studies. Nejatian, Tehrani [39] evaluated and modified the 35-question questionnaire version of the scale. After the evaluation, six questions were removed and the modified version of the MHLS with 29 items and six attributes was confirmed. For the reliability, McDonald's Omega coefficient and Cronbach's alpha coefficient were calculated to be 0.797 and 0.789, respectively.

### Questionnaire 2) mental health General knowledge and e Attitudes to Mental disorders/Illnesses

The mental health general knowledge questionnaire consists of 28 questions responded to on a 3-point scale (“True”, “False”, or “I don't know”) based on the six modules of the guide. The Attitudes to Mental Illness Questionnaire consists of 8 questions including include statements about mental disorders or people with a mental illness and ask respondents to express their level of agreement using a Likert scale (from "strongly disagree" to "strongly agree"). Mcluckie, Kutcher [14]evaluated the mental health general knowledge questionnaire and attitudes to mental illness, and Cronbach's alpha 0/71 was confirmed.

### Data analysis

To describe and analyze data, descriptive and inferential statistical methods are utilized. Qualitative variables will be presented as frequency and percentage, while quantitative variables will be presented as mean and standard deviation. The paired t-test will be used to measure the significant difference in mental health literacy, knowledge, and attitude between the before and after surveys. *p* < 0.05 will be considered as a significant level. SPSS software (version 26) will be used for analysis.

## Discussion

The use of a curriculum-integrated mental health literacy resource (guide) offered by classroom instructors may aid students in understanding mental health and mental illness, eventually resulting in a decrease in the stigma of mental illness and an increase in the number of people seeking treatment. Additionally, it will encourage the development of mental health literacy in teachers and students by improving teachers' and students' attitudes about mental health.

### Strengths and limitations

One of the study's strength is that the Guide will be localized, thereby enabling the substitution of educational content that conflicts with Iranian culture with more suitable material. Another strength is that the educational content of the mental health literacy program will be delivered by school instructors, and its efficacy will align more closely with the program's objectives. From other strengths, this study is a randomized controlled trial with an intervention and a control group, and also the most important point in this study will be the measuring tool of the MHLS questionnaire, which includes all the key components of mental health literacy.

This study also has limitations. The absence of the school-based mental health literacy program guide in the students' curriculum is one of the limitations of this study. Therefore, instructors and students may lack the cooperation necessary for the program's implementation.

## Data Availability

The datasets generated and/or analyzed during the current study are available from the corresponding author on reasonable request.

## Abbreviations

CVI: Content Validity Index
CVR: Content Validity Ratio
SAM: Suitability Assessment of Materials
RAM: Readability Assessment of Materials
MHL_S_: Mental Health Literacy Scale
